# Common Familial Effects on Ischemic Stroke and Myocardial Infarction: A Prospective Population-Based Cohort Study

**DOI:** 10.3389/fcvm.2014.00003

**Published:** 2014-07-30

**Authors:** Katherine Kasiman, Cecilia Lundholm, Sven Sandin, Ninoa Malki, Pär Sparén, Erik Ingelsson

**Affiliations:** ^1^Department of Medical Epidemiology and Biostatistics, Karolinska Institutet, Stockholm, Sweden; ^2^Saw Swee Hock School of Public Health, National University of Singapore, Singapore, Singapore; ^3^Molecular Epidemiology and Science for Life Laboratory, Department of Medical Sciences, Uppsala University, Uppsala, Sweden

**Keywords:** epidemiology, family history, heritability, ischemic stroke, coronary heart disease

## Abstract

**Background:** Recent genome-wide association studies suggest some overlap of genetic determinants of ischemic stroke (IS) and myocardial infarction (MI). This study aimed to assess shared familial risk between IS and MI in a large, population-wide cohort study.

**Methods:** Study participants free of IS and MI and their affected siblings were extracted from the Swedish Hospital Discharge and Cause of Death Registers between 1987 and 2007, forming an exposed sib-pair. They were matched by birth year of both siblings and calendar period to up to five unexposed sib-pairs. Stratified Cox regression analyses were used to assess familial risk of MI and IS in those exposed to having a sibling with IS (*n* = 31,659) and MI (*n* = 62,766), respectively, compared to unexposed (*n* = 143,728 and 265,974).

**Results:** The overall risk of MI when exposed to having a sibling with IS was statistically significantly increased (RR, 1.44; 95% CI, 1.34–1.55, *p* < 0.001) to a similar extent as risk of IS when exposed to having a sibling with MI (RR, 1.41; 95% CI, 1.32–1.50, *p* < 0.001). The familial risks were similar in full siblings for both groups (RR for MI, 1.46; 95% CI, 1.35–1.58, *p* < 0.001; and RR for IS, 1.40; 95% CI, 1.30–1.40, *p* < 0.001) and half siblings (RR for MI, 1.29; 95% CI, 1.05–1.59, *p* < 0.001; and RR for IS, 1.38; 95% CI, 1.16–1.65, *p* < 0.001).

**Conclusion:** This large, population-wide study indicates that there is considerable overlap of familial risk between IS and MI.

## Introduction

Ischemic stroke (IS) and myocardial infarction (MI) both being diseases of vascular nature have overlapping risk factors (1). There is also evidence that IS and MI shares some genetic determinants, but the full extent of this overlap is unknown. The risk allele at the well-known coronary heart disease (CHD) locus, CDKN2A/CDKN2B in 9p21, has been shown to be associated also with IS (2), and a recent genome-wide analysis using data from METASTROKE, Coronary Artery Disease Genome-wide Replication and Meta-analysis (CARDIoGRAM), and Coronary Artery Disease (C4D) Genetics consortia reported 15 loci at genome-wide significant association with both IS and CHD in a joint analysis (3). Moreover, several studies in first-degree relatives have explored the associations between family history of IS and the risk of IS; family history of MI and the risk of MI; or family history of either these diseases and the risk of cardiovascular disease in general (4–16).

However, only a few relatively small studies have systematically looked at the co-inheritance of familial factors of one condition affecting risk of the other (6, 16, 17). A recent population-based study in the United Kingdom compared the relative familial clustering of IS or transient ischemic attack (TIA) versus MI in parent–offspring relations within the same condition showing greater within-family clustering of MI compared to stroke (6). Another community-based study in the United States explored the association between sibling history of MI or stroke, and the risk of cardiovascular disease among elderly individuals. They reported that a composite positive sibling history of MI or stroke was associated with increased cardiovascular disease risk, but did not find evidence of co-inheritance in these diseases (16). To our knowledge, no prior studies have addressed potential effects of sex, age at onset, or sibling kinship on co-inheritance of either IS or MI.

Hence, the goal of our study was to assess and characterize shared familial risk between IS and MI in a large, population-wide matched cohort study. In addition, we wanted to explore and compare shared effects of sibling kinship, sex, and onset age of one disease on the risk of the other.

## Materials and Methods

### Study populations and design

**Figure 1:**
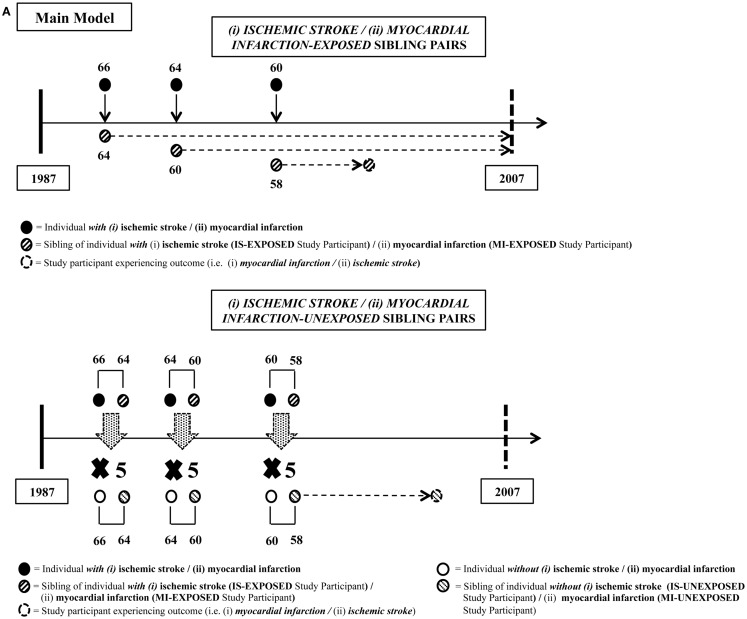

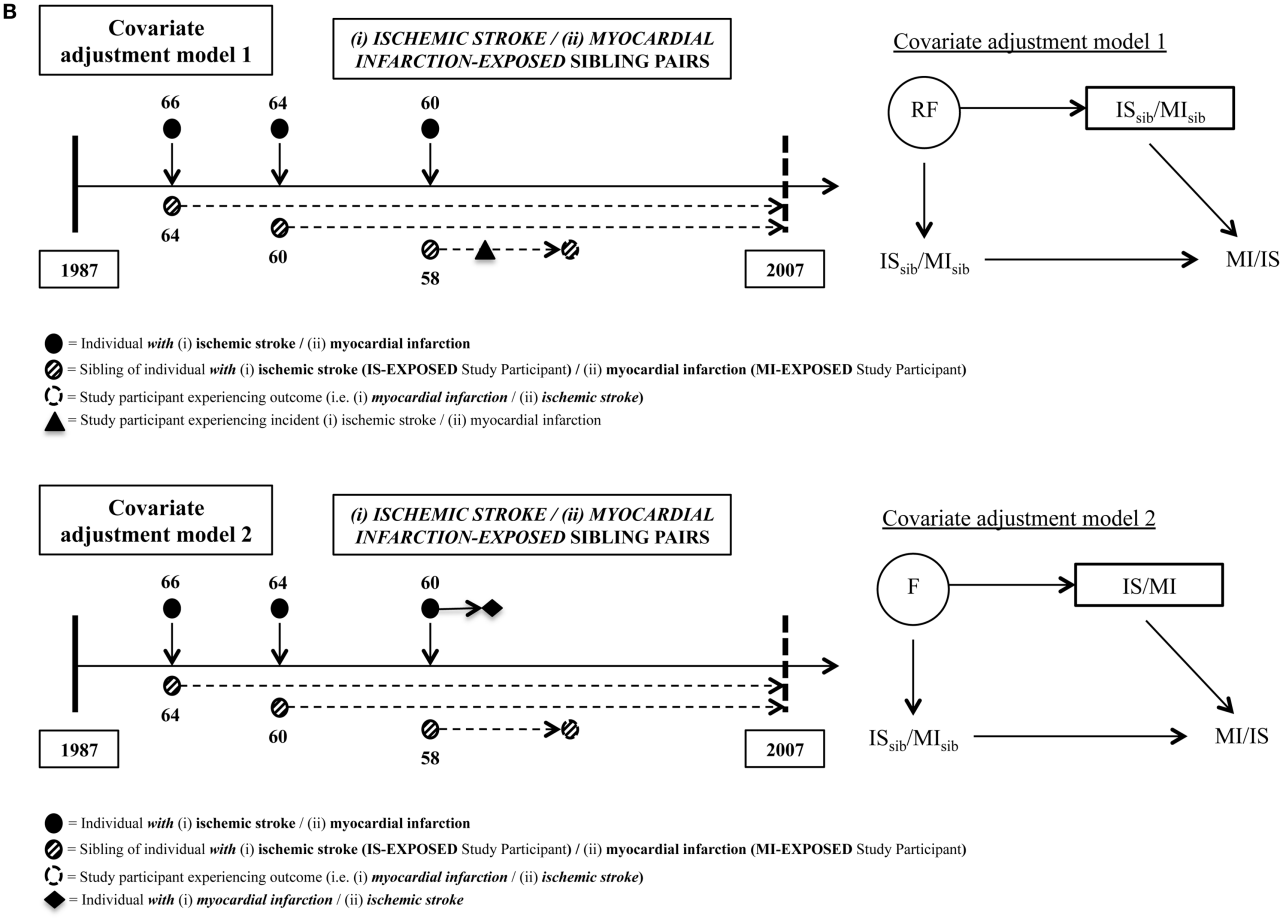
**Details of matching steps for exposed and unexposed sibling pairs for both study populations**. **(A)** Main model (i) myocardial infarction (MI) risk among study participants with siblings who had ischemic stroke (IS) compared to those with siblings who did not have IS, and (ii) IS risk among study participants with siblings who had MI compared to those with siblings who did not have MI. **(B)** Sensitivity analyses models: covariate adjustment model 1 estimates (i) MI risk among study participants taking into account the incidence of IS post-study entrance and prior to MI outcome or end of study among study participants, and (ii) IS risk among study participants taking into account the incidence of MI post-study entrance and prior to IS outcome or end of study among study participants. Time-varying covariate covariate adjustment model 2 estimates (i) MI risk among study participants with siblings who had IS, taking into account subsequent MI post-IS in siblings, compared to those with siblings who did not have both of these conditions, and (ii) IS risk among study participants with siblings who had MI, taking into account subsequent IS post-MI in siblings, compared to those with siblings who did not have both of these conditions. In IS-exposed dataset, covariate adjustment model 1 added “MI” in siblings as the additional covariate to be adjusted, acting as a proxy for adjusting risk factors (RF). Covariate adjustment model 2 added “IS” in study participants occurring prior to MI outcome as the additional covariate to be adjusted, acting as a proxy for adjusting shared familial and environmental risks (F).

The overall design of this study has been described previously in detail (14) and is outlined in Figure 1. Briefly, this population-based sib-pair cohort study utilized the ability of linking various registers in Sweden, mainly the Swedish Multi-Generation Register (MGR), the Hospital Discharge Register (HDR), and the Cause of Death Register (CDR), through the personal identification numbers (PINs) issued to all Swedish residents. Individuals with events or deaths (IS or MI, separately) as primary diagnosis between January 1, 1987 and December 31, 2007 were extracted from the HDR and CDR. Strict definitions of the International Classification of Diseases (ICD) editions 9 (ICD-9) and 10 (ICD-10) were used for both IS (ICD-9: 433–434; ICD-10: I63) and MI (ICD-9: 410; ICD-10: I21–I22) to ensure more accurate estimates in our analyses. Linkage made through their biological parents from the MGR identified one event-free sibling (sibling free of MI for individuals with IS and sibling free of IS for individuals with MI) at the time of diagnosis. These event-free siblings are termed as “exposed study participants” from now on, and they make up the subjects of interest in our study (study participants exposed to having a sibling with IS or MI, respectively). Collectively, these exposed study participants and their respective siblings formed the exposed sib-pair. In this study, an IS-exposed study participant was defined as an MI-free individual having a sibling with IS at baseline, while an MI-exposed study participant was defined as an IS-free individual having a sibling with MI at baseline. Up to five unexposed sib-pairs were randomly selected via matching by birth year of both siblings and event onset age of the sibling, i.e., calendar year, to each exposed sib-pair through the MGR. Unexposed study participants were defined as event-free individuals having a sibling without prior relevant event at the time of inclusion. For example, an MI-unexposed study participant was an IS-free individual having a sibling without MI at the time of inclusion. Using this design, a total of 31,659 IS-exposed and 143,728 IS-unexposed study participants were included in the IS dataset, and a total of 62,766 MI-exposed and 265,974 MI-unexposed study participants were included in the MI dataset. Only residents in Sweden at the time of the study were included in this study. Information on sibling status (full or half), sex, birth date, hospitalizations on MI and IS events, and deaths (including causes of death) were collected. Study participants were followed-up until MI (for IS dataset) or IS (for MI dataset) event, death or end of follow-up period at 31 December 2007, whichever came first.

### Data sources

The HDR is a register collecting data on in-patients treated at public hospitals with a nation-wide coverage since 1987 and onward. This register contains information on dates of admission and discharges with up to eight discharge diagnosis codes, the first representing the principal cause of hospitalization. The CDR, a nation-wide reporting system, documents death and causes of death since 1749. Together with the HDR, the CDR was used to collect information on IS and deaths in our study population. The MGR, a national register consisting of all individuals born from 1932 onward who had been registered in Sweden since 1961, has the ability to connect individuals to their biological and adoptive parents and henceforth siblings through the unique Swedish PINs assigned to all individuals born in Sweden, or who relocate to Sweden for a period of 1 year and longer. These PINs can be used to link data in various national registers, such as the HDR and the CDR. The National Census Data (1990) and the Education Registry (after 1990) were used to collect information on education level (where we further categorized this education level into four main groups for analyses – primary school, secondary-technical school, secondary-theoretical school, and college/university) as a proxy for socioeconomic status. The study was approved by the Regional Ethics Committee Stockholm (2009/940-31/5).

### Statistical analyses

**Figure 2:**
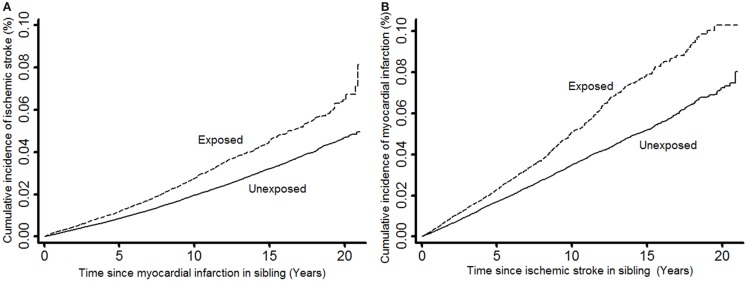
**Cumulative incidence of ischemic stroke in study participants when exposed and unexposed to having a sibling with myocardial infarction (A) and cumulative incidence of myocardial infarction in study participants when exposed and unexposed to having a sibling with ischemic stroke (B)**.

We calculated the time from the entry of study participants (incident IS or MI event in the sibling for exposed or time of matching for unexposed) until study exit (MI or IS events, censoring due to death from other causes, or end of follow-up at 31 December 2007). Primarily, two types of relative risks (RRs) were estimated by calculating the hazard ratios from stratified Cox regression models using matching factors to define strata to account for the matched study design: (1) the RRs of IS comparing MI-exposed study participants (individuals having a sibling with MI) with unexposed study participants (individuals having a sibling without MI) were estimated; and (2) the RRs of MI comparing IS-exposed study participants (individuals having a sibling with IS) with unexposed study participants (individuals having a sibling without IS) (Figure 2A). The RRs and two-sided 95% Wald-type confidence intervals (CIs) of incident IS and MI events in exposed versus unexposed were estimated. For each of MI and IS, incidence over time was illustrated by cumulative incidence plots (Figure 2).

When calculating whether having a sibling with one type of outcome confers a risk of the other outcome, there may be a bias if the sibling experienced both outcomes since this could lead to a false impression of common familial effects. To address this, we carried out sensitivity analyses via two alternate models where the aforementioned main model was adjusted for an additional covariate. In analyses assessing RRs of MI, the two adjusted alternate models were: (1) IS prior to incidence of MI in study participants (in other words, the occurrence of IS before the outcome of interest, which was incidence of MI in study participants) (model 1); and (2) MI in sibling *after* having a sibling with IS (in other words, an additional exposure to sibling risk of MI after being exposed to sibling risk of IS) (model 2) for assessing RRs of MI. In a parallel fashion, in analyses assessing RRs of IS, time-varying covariates were: (1) MI prior to incidence of IS in study participants (in other words, the occurrence of MI before the outcome of interest, which was incidence of IS in study participants) (model 1); and (2) IS post-MI in sibling (in other words, an additional exposure to sibling risk of IS after being exposed to sibling risk of MI) (model 2). These models are delineated in Figure 1B.

To assess the role of familial risk of early IS onset and early MI onset, we also analyzed the risks of IS and MI before the age of 55. The Cox models were also fitted in subgroups stratified by sib-ship (full/half siblings), sex, sex of the sibling, and by onset age of IS or MI in the sibling (early: up to age 55; late: after age 55). Sensitivity analyses following the aforementioned alternate models were also implemented in these sets of analyses.

Proportional hazard assumptions were confirmed using Schoenfeld residuals (18). Two-tailed significance values were given with *p* < 0.05 regarded as statistically significant. Data were analyzed using SAS version 9.2 (SAS Institute Inc., Cary, NC, USA) and STATA for Windows version 11.2 (StataCorp LP, College Station, TX, USA) packages.

### Role of the funding source

This study was supported by the SIMSAM project, a grant from the Swedish Research Council (VR; Grant No. 2008-7483). The study sponsors had no role in study design, data collection, data analysis, data interpretation, or writing of the report. The corresponding author had full access to all the data in the study and had final responsibility for the decision to submit for publication.

## Results

In this study, a total of 31,659 IS-exposed (individuals having a sibling with IS) and 143,728 IS-unexposed (individuals having a sibling without IS) study participants were included, generating a total of 199,582 and 939,337 person-years at risk of follow-up, respectively, for the analyses with MI as outcome. The numbers of MI events during follow-up were 1,011 among the IS-exposed, and 3,294 among the IS-unexposed. Further, a total of 62,766 MI-exposed (individuals having a sibling with MI) and 265,974 MI-unexposed (individuals having a sibling without MI) study participants were included, generating a total of 461,694 and 2,042,377 person-years at risk of follow-up, respectively, for the analyses with IS as outcome. The numbers of IS events during follow-up were 1,314 among the MI-exposed, and 4,130 among the MI-unexposed. The cumulative incidence of MI in our study participants was shown to be much higher than the cumulative incidence of IS regardless of whether our study participants were exposed or unexposed to IS or MI, respectively (Figure 2). Table 1 shows the characteristics of our study participants and their siblings.

**Table 1:** **Characteristics of unexposed and exposed study participants when up to five unexposed sib-pairs were matched to every exposed sib-pair**.

	Exposure: ischemic stroke		Exposure: myocardial infarction	
	Outcome: myocardial infarction		Outcome: ischemic stroke	
	Unexposed	Exposed	Unexposed	Exposed
Full sample, *n* (%)	143,728	31,659	265,974	62,766
Time-at-risk, person-years	939,337	199,582	2,042,377	461,694
Sibling relation, *n* (%)
Full	127,568 (82.4)	27,227 (17.6)	236,372 (81.5)	53,711 (18.5)
Half	16,165 (77.9)	4,578 (22.1)	29,604 (75.6)	9,538 (24.4)
Onset age of outcome event
Mean, years (SD^a^)	63.6 (6.32)	62.9 (6.35)	64.2 (5.98)	64.0 (6.27)
Median, years (IQR^b^)	64.4 (59.6–68.4)	63.8 (59.2–67.6)	64.9 (60.4–68.7)	64.7 (60.2–68.7)
Sex, *n* (%)
Male	71,121 (82.1)	15,509 (17.9)	133,427 (81.2)	30,949 (18.8)
Female	72,612 (81.7)	16,296 (18.3)	132,549 (80.4)	32,300 (19.6)
Education, *n* (%)
Primary	43,282 (81.3)	9,980 (18.7)	73,214 (79.2)	19,229 (20.8)
Secondary – technical	37,946 (81.4)	8,655 (18.6)	65,674 (80.3)	16,134 (19.7)
Secondary – theoretical	12,322 (81.6)	2,785 (18.4)	21,835 (81.1)	5,100 (18.9)
Tertiary	27,614 (83.2)	5,565 (16.8)	49,612 (83.2)	9,997 (16.8)
Unknown	22,559 (82.4)	4,820 (17.6)	55,641 (81.3)	12,789 (18.7)
Birth year, *n* (%)
1932–1939	51,684 (81.3)	11,904 (18.7)	93,246 (79.7)	23,783 (20.3)
1940–1949	63,975 (82.0)	14,093 (18.1)	119,384 (81.0)	28,030 (19.0)
1950–1959	21,665 (82.8)	4,505 (17.2)	43,118 (82.2)	9,327 (17.8)
1960–1969	6,409 (83.1)	1,303 (16.9)	10,228 (82.9)	2,109 (17.1)
Diagnosis year, *n* (%)
1987–1989	4 (50.0)	4 (50.0)	4 (40.0)	6 (60.0)
1990–1999	529 (71.4)	212 (28.6)	710 (71.1)	288 (28.9)
2000–2007	2,762 (76.3)	857 (23.7)	3,416 (75.0)	1,137 (25.0)

*^a^SD denotes standard deviation*.

*^b^IQR denotes inter-quartile range*.

Overall, the study participants who had been exposed to having a sibling with IS were observed to have a ~40% higher risk of MI compared to those who had not been exposed. A similar magnitude of increased risk for IS was also observed in study participants who had been exposed to having a sibling with MI compared to those who had not been exposed. The RR for MI remained similar when IS events in study participants’ post-study entrance were taken into account (Supplementary Table 1, left panel; model 1). However, the RR for MI was reduced to 1.32 (from 1.44) when the model was adjusted for MI in siblings (Supplementary Table 1, left panel; model 2). The RR for IS was reduced to 1.24 (from 1.41) when MI event post-study entrance were taken into account or when the model was adjusted for IS in siblings (Supplementary Table 1, right panel; models 1 and 2).

**Figure 3:**
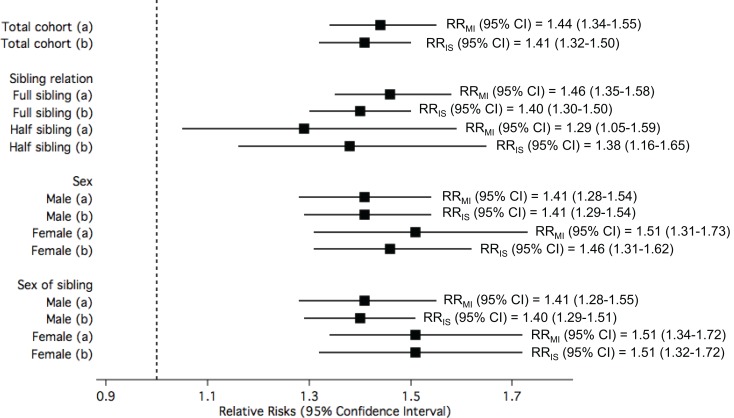
**The relative risks (RRs) of (a) myocardial infarction (MI) among study participants when exposed to siblings with ischemic stroke (IS) compared to unexposed; and (b) IS among study participants when exposed to siblings with MI compared to unexposed, in the total cohorts and stratified by sibling relation, sex of study participants, and sex of siblings**.

Ischemic stroke-exposed full siblings had higher RR of MI compared to IS-exposed half siblings. However, this difference in RR among sibling relation was not statistically significant. On the other hand, MI-exposed full siblings had similar RR of IS compared to MI-exposed half siblings. A pattern of higher risk in women compared to men was consistently observed when the analyses were stratified by sex of study participants and sex of their siblings; however, the differences were not statistically significant (Figure 3).

**Figure 4:**
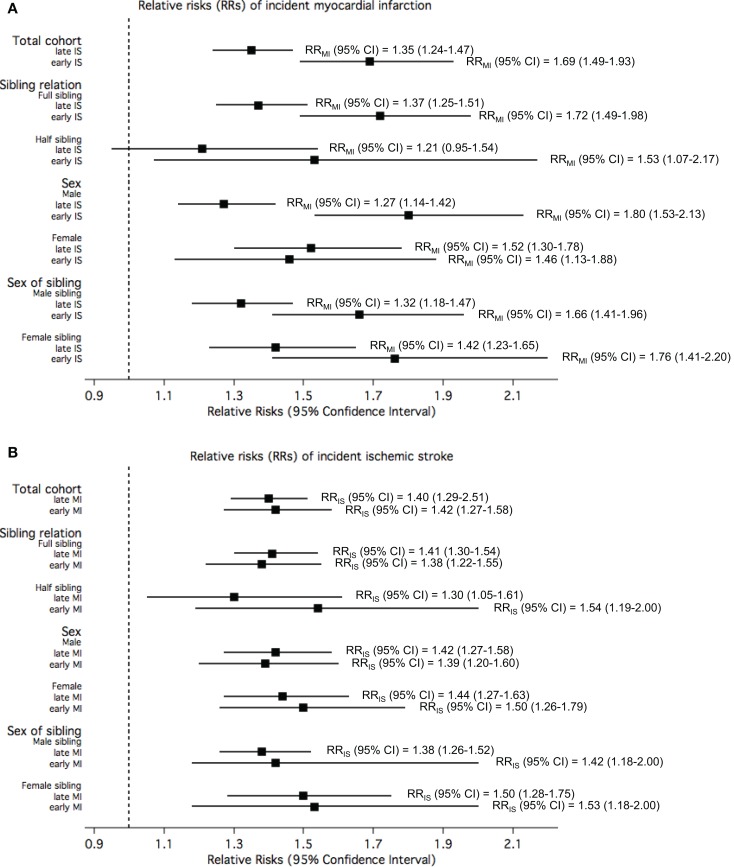
**The relative risks (RRs) of (A) myocardial infarction (MI) among study participants when exposed to siblings with different onset age of ischemic stroke (IS) compared to unexposed; and (B) IS among study participants when exposed to siblings with different onset age of MI compared to unexposed, in the total cohorts and stratified by sibling relation, sex of study participants, and sex of siblings**.

Figure 4A displays the RRs of MI in the study participants when the exposure was defined according to their siblings’ age of IS onset. Overall, the risk of MI in the study participant was statistically significantly elevated by 70% when their siblings had early IS compared to when their siblings did not have IS. This risk was statistically significantly higher than the risk of MI among participants with siblings with late-onset IS. The pattern of statistically significantly higher RR for early-onset compared with late onset was true also when stratified by sex of sibling and sibling relation. The increased risk of IS in the study participant when exposed to siblings with early or late-onset MI compared to unexposed was similar, and this similarity in risk estimates between the two groups of onset age as exposure was observed when stratified by sex, sex of sibling, and sibling relation (Figure 4B).

**Figure 5:**
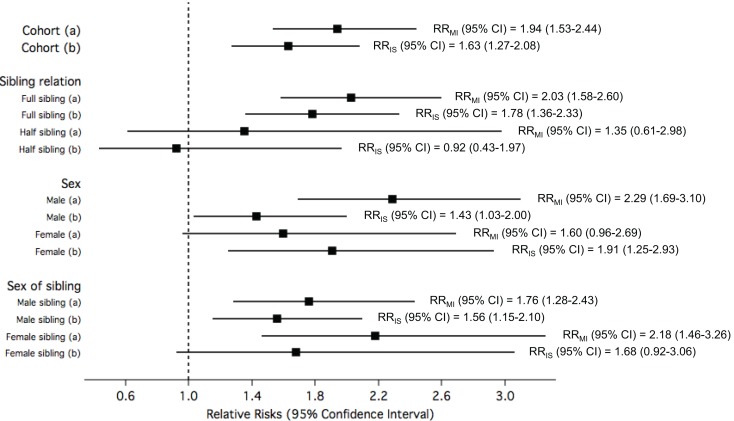
**The relative risks (RRs) of (a) *early* myocardial infarction (MI) among study participants when exposed to siblings with *early* ischemic stroke (IS) compared to unexposed; and (b) *early* IS among study participants when exposed to siblings with *early* MI compared to unexposed, in the total cohorts and stratified by sibling relation, sex of study participants, and sex of siblings**.

Figure 5 shows the RRs for early-onset MI or IS among study participants exposed to siblings with early-onset IS or MI, respectively, compared to unexposed. Overall, the risk for early MI among study participants exposed to siblings with early IS was observed to be 94% higher than those whose siblings did not have IS, while the risk for early IS among study participants exposed to siblings with early MI when compared to those with siblings without MI was slightly lower (RR = 1.63). Stratification by sibling relation, sex, and sex of sibling showed that the RRs for early-onset MI among study participants exposed to early IS compared to unexposed approximately doubled in full sibling relations, males, and female siblings. The RRs for early-onset IS among study participants exposed to early MI compared to unexposed were not as high, and stratification by sibling relation, sex, and sex of sibling showed higher risk among full siblings relations, females, and female siblings.

All the rates, RRs, their corresponding *p*-values and *p* for interactions are tabulated in the Supplementary Material (Supplementary Tables 1 and 2 for Figure 3; Supplementary Table 3 for Figure 4; and Supplementary Table 4 for Figure 5).

## Discussion

In this study of common familial effects of IS and MI taking the role of sibling kinship, sex, and age of onset into account, we found that among 31,659 IS-exposed and 143,728 IS-unexposed study participants from all over Sweden, having a sibling with IS increased the risk of having a MI by 44% compared to having a sibling without IS. Similarly, among 62,766 MI-exposed and 265,974 MI-unexposed study participants, having a sibling with MI increased the risk of having an IS by 41% compared to having a sibling without MI. There was evidence for stronger familial effects for early-onset disease, but no consistent effects of sex or sibling kinship.

Additional covariate adjustment analyses showed that the risk of MI associated with having a sibling with IS was not affected by having an IS yourself prior to the MI. In contrast, the risk of IS associated with having a sibling with MI was attenuated (albeit still statistically significant) when taking interim MI in the study participant into account. Further, taking interim events in siblings into account attenuated the sibling risks of both MI and IS. These observations taken together suggest that genetic factors related to MI are contributing toward risk of both conditions in a stronger fashion than genetic factors related to IS. This is consistent with another study whereby heritability of coronary events was found to be greater than heritability of cerebral events (6). A recent study by Siegerink et al. also found that a family history of MI was more frequent in MI cases compared with IS cases. They showed that neither a family history of MI nor of stroke were strong predictors of IS, whereas both were associated with MI, suggesting that familial clustering of MI is more prominent than familial clustering of IS (17).

Our results are not consistent with results from the Cardiovascular Health Study, where the MI risk was neither associated with a positive sibling history for stroke (composite of IS/TIA) nor was the risk of stroke higher with a positive history for MI (16). The report from the Cardiovascular Health Study included 5,888 elderly individuals, all stroke subtypes and utilized self-reported sibling history. In contrast, our study was based on a much larger sample size, had more stringent disease definitions and physician-validated sibling history; these differences may explain the discrepant results.

Sex effects have been explored in previous studies of familial aggregation of either stroke or CHD. One prior study assessed the importance of sex-of-parent/sex-of-proband offspring interactions in the family history of MI in acute coronary syndrome patients (5), reporting that maternal history of MI was twice as common in women with early acute coronary syndrome, as in men with early acute coronary syndrome. Previous population-based studies in stroke also emphasized that women are more likely than men to have a history of stroke among female first-degree relatives suggesting sex-specific transmission of stroke. In a separate study, Banerjee and colleagues also found maternal stroke to be twice as common as paternal stroke in women with acute coronary syndrome, but not in men with the same condition, and that women with acute coronary syndrome were more likely to have stroke-affected female first-degree relatives compared to men with acute coronary syndrome having stroke-affected male first-degree relatives (4). In our study, the risk of MI and IS were increased in similar magnitudes as the overall risk in each disease in both sexes and sexes of siblings. Notably, all RRs seemed to be slightly higher among women compared to men, but these differences were modest and not statistically significant. This suggested the absence of sex differences in the lifetime-shared risk of both conditions.

Having a sibling with early-onset of either IS or MI increased the risk for the study participant to have an early-onset of MI or IS, respectively, although the magnitude of increase differed slightly. This was expected as our previous study and other studies on familial aggregation of stroke found associations with younger age of onset within the same condition (14, 19, 20). Moreover, many common risk factors have been strongly associated with familial risk of both conditions, which further strengthened this assumption (1).

Cardiovascular risk factors such as hypertension, smoking, diabetes, obesity, hypercholesterolemia, unhealthy diet, and low physical activity are associated with development of both IS and MI, and they also have substantial genetic components with heritabilities estimated in twin studies ranging from ~30 to 70% (21–25). Hence, it is likely that these factors could act as mediators of the observed associations, but the extent to which such mediation occurs as a result of genetic factors or shared environment cannot be disentangled using the present study design. One of the most important risk factors and possible mediators is hypertension. Observational studies including more than 1 million individuals have established that increased blood pressure is associated with similar magnitudes of risk increase of IS and MI; for every 20 mmHg systolic or 10 mmHg diastolic blood pressure increase (from 115/75 mmHg), there is a doubling of mortality from both IS and MI (26). The heritabilities of systolic and diastolic blood pressure have been reported to be 44–71 and 34–52%, respectively, in Northern European populations (21, 27, 28). Indeed, there is some evidence of overlap of genetic determinants of hypertension, MI, and IS. Of the 46 genome-wide significant genetic variants in a study by the CARDIoGRAMplusC4D consortium, 5 were also significantly associated with blood pressure (29). Although twin studies (21, 27, 28) do not provide evidence for a strong shared environmental component in the pathophysiology of hypertension (as the variance is explained by the additive genetic and non-shared environment components), one still cannot definitely exclude shared environment as a partial explanation of our findings. For example, preeclampsia or gestational hypertension predisposes to later life hypertension in offspring (30), which in turn increase their risk of IS or MI. As the risk for preeclampsia or gestational hypertension in subsequent pregnancies is increased (31), this could result in a shared environmental risk factor among siblings or common genetic factors predisposing to increased blood pressure (32).

The strengths of our study include the publicly available and financed Swedish health system, which gives nation-wide coverage, resulting in a very large, representative sample, allowing us to study the familial risks of IS and MI taking sibling relations, sex of siblings, and age of onset into account. The prospectively collected data, the non-differential follow-up of both IS incidence and mortality, and the inclusion of only IS instead of all strokes (which include hemorrhagic strokes and TIAs that are likely to be less relevant for co-heritability with MI) are other important strengths of this study. Moreover, sibling history of IS and MI were physician-validated. Many previous studies relied on self-reported questionnaires to obtain medical history of siblings making it prone to ascertainment bias, and they also were unable to identify sibling relations in their study population. However, there are also several limitations to our study. Due to truncation of the register data, inclusion into the cohort was conditional of (1) sib-pair being alive after 1987; and (2) index sibling not being older than 55 years in 1987 (since they would have been born before 1932). As a consequence, age at diagnosis is likely to be confounded by calendar time, since the proportion of older individuals in this study increase from 1987 to 2007. However, this potential limitation was dealt with by taking both age and calendar time into account in the study design. Further, subjects with IS or MI events occurring before 1987 could be falsely classified as healthy (since the coverage of the register was not complete until 1987); thereby diluting a real recurrence ratio of more than one, inadvertently driving the associations toward the null (underestimating the real effects). Finally, we did not have information on stroke subtypes within IS or specific type of MI (ST-elevation or non-ST-elevation), which may be important in the heritability of IS and MI.

Our findings could have a considerable public health importance in the education about IS and MI in the general population, and they may be relevant for the practicing clinicians when advising patients with first-degree relatives with prior IS or MI. Sibling history seems to be as important as parental history in these conditions, and perhaps more relevant as siblings typically share many dietary and lifestyle factors on top of genetics, and since sibling history may be easier to obtain.

In conclusion, there was a shared familial aggregation between IS and MI estimated to a 44% increased risk for MI in individuals having a sibling with prior IS, and a 41% increased risk for IS in individuals having a sibling with prior MI.

## Author Contributions

All authors contributed to the study design, data analysis and interpretation, writing of the manuscript, and reviewed the final version for publication.

## Conflict of Interest Statement

The authors declare that the research was conducted in the absence of any commercial or financial relationships that could be construed as a potential conflict of interest.

## Supplementary Material

The Supplementary Material for this article can be found online at http://www.frontiersin.org/Journal/10.3389/fcvm.2014.00003/abstract
