# Supplementary material for: Common Familial Effects on Ischemic Stroke and Myocardial Infarction: A Prospective Population-Based Cohort Study
Source: Front Cardiovasc Med. 2014 Jul 30;1:3. doi: 10.3389/fcvm.2014.00003 (PMC4668847; doi:10.3389/fcvm.2014.00003)
Supplement: Supplementary file 1 [file Presentation1.PDF]

Supplementary Table 1: Rates and relative risks (RRs) of (a) myocardial infarction (MI) among study participants when exposed to siblings with ischemic stroke (IS) compared to unexposed; and (b) IS among study participants when exposed to siblings with MI compared to unexposed

	(a) Sibling with IS -> Risk for MI				(b) Sibling with MI -> Risk for IS			
	**No of events/N**	**Rate per 1000 person-years (95% CI[*])**	**RR (95% CI[*])**	**p-value**	**No of events/N**	**Rate per 1000 person-years (95% CI[*])**	**RR (95% CI[*])**	**p-value**
**Main model**								
Unexposed	3,294/ 143,728	3.51 (3.39-3.63)	1.44 (1.34-1.55)	**<0.001**	4,130/ 265,974	2.02 (1.96-2.08)	1.41 (1.32-1.50)	**<0.001**
Exposed	1,011/ 31,659	5.07 (4.76-5.39)			1,314/ 62,766	2.85 (2.70-3.00)		
**Covariate adjustment model 1**								
**Covariate = IS in study participant**					**Covariate = MI in study participant**			
Unexposed	3,044/ 133,434	3.48 (3.35-3.60)	1.44 (1.33-1.55)	**<0.001**	3,669/ 235,154	2.03 (1.96-2.10)	1.24 (1.15-1.33)	**<0.001**
Exposed	949/ 28,621	5.06 (4.75-5.39)			1,085/ 52,480	2.65 (2.49-2.81)		
**Covariate adjustment model 2**								
**Covariate = MI in sibling**					**Covariate = IS in sibling**			
Unexposed	3,016/ 132,538	3.46 (3.34-3.59)	1.32 (1.22-1.42)	**<0.001**	3,718/ 236,904	2.04 (1.98-2.11)	1.24 (1.15-1.33)	**<0.001**
Exposed	906/	4.95			1,073/	2.63		

	27,760	(4.64-5.28)			52,304	(2.47-2.79)		
*CI denotes confidence interval								

Supplementary Table 2: Rates and relative risks (RRs) of (a) myocardial infarction (MI) among study participants when exposed to siblings with ischemic stroke (IS) compared to unexposed when stratified by sibling relation, sex, and sex of sibling; and (b) IS among study participants when exposed to siblings with MI compared to unexposed when stratified by sibling relation, sex, and sex of sibling

	(a) Sibling with IS -> Risk for MI					(b) Sibling with MI -> Risk for IS				
	**No of events /N**	**Rate per 1000 person-years (95% CI*)**	**RR (95% CI*)**	**p-value**	**Interaction p-value**	**No of events/ N**	**Rate per 1000 person-years (95% CI*)**	**RR (95% CI*)**	**p-value**	**Interaction p-value**
**<u>By sibling relation</u>**										
Full sibling					0.2820					0.9118
Unexposed	2,857/ 127,565	3.43 (3.31-3.56)	1.46 (1.35-1.58)	**<0.001**		3,551/ 236,370	1.96 (1.90-2.02)	1.40 (1.30-1.50)	**<0.001**	
Exposed	859/ 27,100	5.05 (4.72-5.39)				1,084/ 53,288	2.81 (2.64-2.98)			
Half sibling										
Unexposed	437/ 16,163	4.10 (3.74-4.51)	1.29 (1.05-1.59)	**0.015**		579/ 29,604	2.52 (2.32-2.74)	1.38 (1.16-1.65)	**<0.001**	
Exposed	152/ 4,559	5.18 (4.42-6.08)				230/ 9,478	2.85 (2.68-3.47)			
**<u>By sex</u>**										

Male					0.4498					0.6189
Unexposed	2,389/ 71,118	5.16 (4.96-5.38)	1.41 (1.28-1.54)	**<0.001**		2,649/ 133,426	2.60 (2.50-2.70)	1.41 (1.29-1.54)	**<0.001**	
Exposed	721/ 15,417	7.47 (6.95-8.04)				805/ 30,633	3.58 (3.34-3.83)			
Female										
Unexposed	905/ 72,610	1.90 (1.78-2.03)	1.51 (1.31-1.73)	**<0.001**		1,481/ 132,548	1.45 (1.38-1.53)	1.46 (1.31-1.62)	**<0.001**	
Exposed	290/ 16,242	2.81 (2.51-3.16)				230/ 9,478	2.15 (1.97-2.35)			
**<u>By sex of sibling</u>**										
Male					0.3904					0.3613
Unexposed	1,641/ 71,286	3.50 (3.33-3.67)	1.41 (1.28-1.55)	**<0.001**		1,963/ 129,899	1.96 (1.87-2.05)	1.40 (1.29-1.51)	**<0.001**	
Exposed	650/ 20,423	4.97 (4.60-5.37)				1,012/ 47,789	2.78 (2.62-2.96)			
Female										
Unexposed	1,653/ 72,442	3.52 (3.35-3.69)	1.51 (1.34-1.72)	**<0.001**		2,167/ 136,075	2.08 (2.00-2.17)	1.51 (1.32-1.72)	**<0.001**	

*CI denotes confidence interval

Supplementary Table 3: Rates and relative risks (RRs) of (a) myocardial infarction (MI) among study participants when exposed to siblings with ischemic stroke (IS) at different age of onset compared to unexposed; and (b) IS among study participants when exposed to siblings with MI at different age of onset compared to unexposed, stratified by sex of study participants, sex of siblings, and sibling relation

	**(a) Sibling with IS -> Risk for MI**						**(b) Sibling with MI -> Risk for IS**				
	**No of events/N**	**Rate per 1000 person-years (95% CI[*])**	**RR (95% CI[*])**	**p–value**	**Interaction p–value**		**No of events/N**	**Rate per 1000 person-years (95% CI[*])**	**RR (95% CI[*])**	**p–value**	**Interaction p–value**
**<u>Full sample</u>**											
No stroke	3,294/ 143,728	3.51 (3.39-3.63)	Ref	—	--	No MI	4,130/ 265,974	2.02 (1.96-2.08)	Ref	—	--
Late stroke[†]	700/ 23,120	5.90 (5.48-6.36)	1.35 (1.24-1.47)	**<0.001**		Late MI[†]	867/ 41,529	3.63 (3.40-3.88)	1.40 (1.29-1.51)	**<0.001**	
Early stroke[‡]	311/ 8,539	3.84 (3.44-4.29)	1.69 (1.49-1.93)	**<0.001**		Early MI[‡]	447/ 21,237	2.00 (1.83-2.20)	1.42 (1.27-1.58)	**<0.001**	
**<u>By sex</u>**											
Male					0.0598						0.7841
No stroke	2,389/ 71,118	5.16 (4.96-5.38)	Ref	—		No MI	2,649/ 133,426	2.60 (2.50-2.70)	Ref	—	
Late strok	484/	8.59	1.27 (1.1	**<0.0**		Late	523/	4.60	1.42 (1.27	**<0.0**	

e†	11,121	(7.86-9.39)	4-1.42)	**01**		MI†	19,895	(4.22-5.01)	-1.58)	**01**	
Early stroke‡	237/4,296	5.91 (5.20-6.71)	1.80 (1.53-2.13)	**<0.001**		Early MI‡	282/10,738	2.52 (2.26-2.85)	1.39 (1.20-1.60)	**<0.001**	
Female											
No stroke	905/72,610	1.90 (1.78-2.03)	Ref	—		No MI	1,481/132,548	1.45 (1.38-1.53)	Ref	—	
Late stroke†	216/11,999	3.47 (3.04-3.97)	1.52 (1.30-1.78)	**0.000**		Late MI†	344/21,634	2.75 (2.48-3.06)	1.44 (1.27-1.63)	**<0.001**	
Early stroke‡	74/4,243	1.81 (1.44-2.27)	1.46 (1.13-1.88)	**0.004**		Early MI‡	165/10,499	1.48 (1.27-1.72)	1.50 (1.26-1.79)	**<0.001**	
**<u>By sex of sibling</u>**											
Male											
No stroke	1,641/71,286	3.50 (3.33-3.67)	Ref	—		No MI	1,963/129,899	1.96 (1.87-2.05)	Ref	—	
Late stroke†	450/14,961	5.72 (5.21-6.27)	1.32 (1.18-1.47)	**<0.001**	0.6846	Late MI †	640/30,798	3.51 (3.25-3.79)	1.38 (1.26-1.52)	**<0.001**	0.6410
Early stroke‡	200/5,462	3.84 (3.34-4.41)	1.66 (1.41-1.96)	**<0.001**		Early MI ‡	372/16,991	2.05 (1.85-2.27)	1.42 (1.18-2.00)	**<0.001**	

Female											
No stroke	1,653/ 72,442	3.52 (3.35-3.69)	Ref	—		No MI	2,167/ 136,075	2.08 (2.00-2.17)	Ref	—	
Late stroke†	250/ 8,159	6.27 (5.53-7.09)	1.42 (1.23-1.65)	**<0.001**		Late MI†	227/ 10,731	4.04 (3.54-4.60)	1.50 (1.28-1.75)	**<0.001**	
Early stroke‡	111/ 3,077	3.85 (3.20-4.64)	1.76 (1.41-2.20)	**<0.001**		Early MI‡	75/ 4,246	1.80 (1.43-2.25)	1.53 (1.18-2.00)	**0.002**	
**<u>By sibling relation</u>**											
Full sibling											
No stroke	2,857/ 127,565	3.43 (3.31-3.56)	Ref	—		No MI	3,551/ 236,370	1.96 (1.90-2.02)	Ref	—	
Late stroke†	595/ 19,688	5.94 (5.48-6.43)	1.37 (1.25-1.51)	**<0.001**		Late MI†	728/ 35,484	3.61 (3.35-3.88)	1.41 (1.30-1.54)	**<0.001**	
Early stroke‡	264/ 7,412	3.77 (3.34-4.25)	1.72 (1.49-1.98)	**<0.001**	0.5662	Early MI‡	356/ 17,804	1.93 (1.74-2.14)	1.38 (1.22-1.55)	**<0.001**	0.5408
Half sibling											
No stroke	437/ 16,163	4.10 (3.74-4.51)	Ref	—		No MI	579/ 29,604	2.52 (2.32-2.74)	Ref	—	
Late stroke†	105/ 3,432	5.71 (4.72-	1.21 (0.95-1.54	0.116		Late MI†	139/ 6,045	3.79 (3.21-	1.30 (1.05-	**0.015**	

		6.92)	)					4.48)	1.61)		
Early stroke[‡]	47/ 1,127	4.29 (3.23-5.72)	1.53 (1.07-2.17)	**0.018**		Early MI[‡]	91/ 3,433	2.36 (1.92-2.89)	1.54 (1.19-2.00)	**0.001**	

*CI denotes confidence interval

†>55 years (age of onset of the affected siblings at study enrolment)

‡<=55 years (age of onset of the affected siblings at study enrolment)

Supplementary Table 4: Rates and relative risks (RRs) of early (a) myocardial infarction (MI) among study participants when exposed to siblings with early ischemic stroke (IS) compared to unexposed; and (b) IS among study participants when exposed to siblings with early MI compared to unexposed, stratified by sex of study participants, sex of siblings, and sibling relation

	(a) Sibling with early IS -> Risk for early MI						(b) Sibling with early MI -> Risk for early IS				
	**No of events/N**	**Rate per 1000 person-years (95% CI[*])**	**RR (95% CI[*])**	**p–value**	**Overall p–value**		**No of events/N**	**Rate per 1000 person-years (95% CI[*])**	**RR (95% CI[*])**	**p–value**	**Overall p–value**
**Full sample**[†]											
No stroke	256/41,347	1.26 (1.11-1.42)	1.94 (1.53-2.44)	**<0.001**	—	No MI	251/99,177	0.52 (0.46-0.59)	1.63 (1.27-2.08)	**<0.001**	—
Early stroke‡	100/8,537	2.41 (1.98-2.93)				Early MI‡	86/21,235	0.85 (0.69-1.05)			
**By sibling relation**											
Full sibling					0.3411						0.1130
No stroke	223/37,154	1.22 (1.07-1.39)	2.03 (1.58-2.60)	**<0.001**		No MI	220/88,830	0.51 (0.45-0.58)	1.78 (1.36-2.33)	**<0.001**	
Early stroke‡	90/7,410	2.49 (2.02-3.06)				Early MI‡	75/17,802	0.89 (0.71-1.12)			
Half sibling											
No stroke	33/4,193	1.63 (1.16-2.30)	1.35 (0.61-	0.4644		No MI	31/10,347	0.62 (0.44-0.88)	0.92 (0.43-	0.840	

Early stroke‡	10/ 1,127	1.87 (1.00-3.47)	2.98)			Early MI‡	11/ 3,433	0.63 (0.35-1.13)	1.97)		
**By sex**											
Male					0.2608						0.3231
No stroke	199/ 20,823	1.94 (1.69-2.22)	2.29 (1.69-3.10)	**<0.001**		No MI	160/ 50,533	0.65 (0.56-0.76)	1.43 (1.03-2.00)	**0.035**	
Early stroke‡	79/ 4,296	3.84 (3.08-4.79)				Early MI‡	53/ 10,738	1.04 (0.80-1.36)			
Female											
No stroke	57/ 20,524	0.57 (0.44-0.73)	1.60 (0.96-2.69)	0.073		No MI	91/ 48,644	0.38 (0.31-0.47)	1.91 (1.25-2.93)	**0.003**	
Early stroke‡	21/ 4,241	1.00 (0.65-1.54)				Early MI‡	33/ 10,497	0.65 (0.46-0.92)			
**By sex of sibling**											
Male					0.4499						0.8356
No stroke	143/ 20,911	1.38 (1.17-1.63)	1.76 (1.28-2.43)	**0.001**		No MI	132/ 49,739	0.54 (0.46-0.64)	1.56 (1.15-2.10)	**0.004**	
Early stroke‡	61/ 5,460	2.38 (1.85-3.06)				Early MI‡	71/ 16,989	0.87 (0.69-1.10)			
Female											
No stroke	113/ 20,436	1.13 (0.94-1.36)	2.18 (1.46-	**<0.001**		No MI	119/ 49,438	0.50 (0.42-0.60)	1.68 (0.92-	0.092	

Early stroke‡	39/ 3,077	2.46 (1.80-3.36)	3.26)			Early MI‡	15/ 4,246	0.75 (0.45-1.24)	3.06)		

*CI denotes confidence interval

†Study population used for analyses in this table consists of a subset of individuals <=55 years at study enrolment drawn from the original study population

‡<=55 years (age of onset of the affected siblings at study enrolment
